# Constructing p‐d Orbital Hybridization via In Situ Generation of Bi_19_Br_3_S_27_/NiS*_x_* for Highly Selective Photothermal CO_2_ Reduction to C_2_H_4_

**DOI:** 10.1002/anie.1626004

**Published:** 2026-09-15

**Authors:** Guangmei Gan, Zhixiong Yang, Mengying Wang, Yang Shi, Yuan Li, Gaoke Zhang, Jie Wu

**Affiliations:** ^1^ Hubei Key Laboratory of Mineral Resources Processing and Environment, Key Laboratory of Green Utilization of Critical Non‐metallic Mineral Resources, Ministry of Education, State Key Laboratory of Silicate Materials for Architectures Wuhan University of Technology Wuhan People's Republic of China; ^2^ Department of Chemistry National University of Singapore Singapore Singapore

**Keywords:** CO_2_ reduction, ethylene, high selectivity, orbital hybridization, photothermal catalysis

## Abstract

Photothermal catalytic CO_2_ reduction to multi‐carbon products is a promising approach for carbon utilization, yet the design of active sites that effectively promote multistep C─C coupling toward selective C_2_H_4_ formation remains a significant challenge. Hence, we employ an in situ interfacial engineering strategy to construct crystalline/amorphous Bi_19_Br_3_S_27_/NiS*_x_* (BBSN) photothermal catalysts with strong p‐d orbital hybridization. The optimized BBSN‐8 achieves a C_2_H_4_ production rate of 41.38 µmol g^−1^ h^−1^ with a selectivity of 96.8% under full‐spectrum irradiation, which exhibits a remarkable advantage in the field of C_2_H_4_ production. Experimental results and theoretical calculations demonstrate that Ni incorporation optimizes CO_2_ adsorption from overly strong Bi─C single‐site binding to balanced multi‐sites adsorption, while newly formed p‐d orbital hybridization establishes electronically cooperative Bi─S─Ni active sites stabilizing both carbon‐ and oxygen‐centered species, synergistically facilitating *CO─CHO* coupling and boosting C_2_H_4_ formation. This work demonstrates that in situ interfacial orbital hybridization can regulate intermediate coupling and steer photothermal CO_2_ reduction toward highly selective C_2_H_4_ formation.

## Graphical abstract

An in situ crystalline/amorphous Bi_19_Br_3_S_27_/NiS*_x_* interface unlocks orbital hybridization‐driven CO_2_ conversion. Newly formed p‐d coupling reshapes the reaction pathway, promotes C─C coupling, and steers photo‐thermal CO_2_ reduction toward highly selective C_2_H_4_ production.

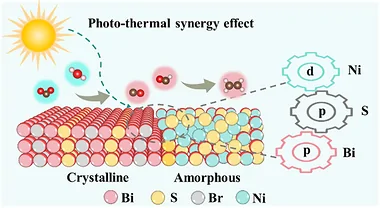

## 1 Introduction

The conversion of CO_2_ into value‐added multi‐carbon products is widely regarded as a promising strategy for mitigating carbon emissions while addressing energy demands [1, 2, 3]. Among various products, ethylene (C_2_H_4_) holds particular importance due to its pivotal role in the chemical industry. Recently, photothermal catalysis has emerged as an effective approach to drive CO_2_ conversion by integrating photo‐induced carrier excitation with thermally accelerated surface reactions [4, 5]. However, the conversion of CO_2_ to C_2_H_4_ over photothermal catalysts still suffers from limited selectivity, largely due to the involvement of complex multi‐electron transfer processes and the requirement for dynamic C─C coupling steps [6, 7, 8]. To address this challenge, considerable efforts have been devoted to strategies such as band structure tuning and defect engineering, which have enhanced charge separation and adsorption properties [9, 10]. Despite these advances, achieving cooperative activation of multiple intermediates during C─C coupling remains difficult. In particular, the formation of C_2_ products requires electronically differentiated active sites that could selectively interact with distinct moieties of reaction intermediates, which is difficult to establish within uniform coordination environment [11, 12].

Constructing electronic coupling between adjacent active centers is expected to enable coordinated interaction with reaction intermediates. Orbital‐level interactions provide an effective means to regulate such coupling by controlling how electrons are distributed and transferred during the reaction [13, 14]. Among various orbital interactions, p‐d orbital hybridization between ligand p orbitals and transition‐metal d orbitals is particularly attractive as it can simultaneously facilitate CO_2_ activation and regulate the evolution of carbon‐containing intermediates through synergistic σ‐donation and π‐backdonation processes [15, 16]. Such orbital coupling could induce molecular polarization and bending of CO_2_, promoting electron injection into CO_2_ antibonding orbitals and thus facilitating the formation of key intermediates [17, 18]. For example, lattice strain engineering in BiFeO_3_ has been shown to optimize d/p‐2π* orbital hybridization, thereby enhancing CO_2_ activation and promoting *COOH intermediates formation [19]. More importantly, p‐d orbital hybridization may further regulate the adsorption and evolution behaviors of carbon‐containing intermediates, thereby influencing C─C coupling kinetics and the product selectivity. Notably, the p‐d orbital hybridization in p‐block metal‐doped Cu catalysts was found to optimize *CO binding and facilitate *CO dimerization, thereby selectively directing CO_2_ electroreduction toward C_2+_ products with a Faradaic efficiency of 81.5% [20]. Hence, the interaction between ligand p orbitals and transition metal d orbitals is expected to achieve highly selective generation of C_2_ products. Nevertheless, achieving efficient p‐d orbital hybridization generally requires strongly coupled interfacial electronic interactions to ensure continuous electron migration and effective orbital overlap [21, 22]. Therefore, employing suitable strategies to construct highly coupled interfaces is essential for maximizing the benefits of p‐d orbital hybridization. In situ construction provides an effective approach for generating strongly coupled heterogeneous interfaces, which promotes the formation of an integrated bonding environment while suppressing electron isolation domains [23, 24]. However, the rational construction of effective p‐d hybridization systems via in situ construction strategy is rarely reported.

Herein, we employ an in situ interfacial engineering strategy to construct Bi_19_Br_3_S_27_/NiS*_x_* photothermal catalyst featuring p–d orbital hybridization for highly selective CO_2_‐to‐C_2_H_4_ conversion. The in situ generated NiS*_x_* induces a balanced multi‐sites adsorption for CO_2_ and constructs the interfacial orbital hybridization. Various results of experimental and characterization tests reveal that the newly formed p‐d orbital hybridization promotes Bi─S─Ni cooperative interactions with CO_2_ molecules, thereby facilitating selective C─C coupling toward C_2_ pathway. As a result, the optimized BBSN‐8 catalyst achieves a high C_2_H_4_ selectivity of 96.8% under full‐spectrum light irradiation. This study not only provides new insights into the role of orbital hybridization in regulating complex reaction pathways but also offers a rational strategy for designing advanced catalysts for multi‐carbon product generation from CO_2_.

## 2 Results and Discussion

### 2.1 Structural and Morphological Properties

The crystalline/amorphous Bi_19_Br_3_S_27_/NiS*_x_* (BBSN) was constructed through an in situ growth strategy, where amorphous NiS*_x_* was in situ generated on crystalline Bi_19_Br_3_S_27_ (BBS) nano‐needles under sulfur‐rich hydrothermal conditions, forming an intimately coupled interface (Figure 1a). The obtained samples were denoted as BBSN‐*x*, where *x* represents the molar ratio of Ni precursor to Bi during preparation. The actual Ni/Bi ratios determined by inductively coupled plasma optical emission spectroscopy (ICP‐OES) are summarized in Table S1. The morphology and microstructure of BBSN were observed by transmission electron microscopy (TEM) characterizations. As displayed in Figure 1b, pristine BBS exhibits smooth‐tipped nanoneedle structures. The interplanar spacing shown in Figure S1 is 0.294 nm and 0.532 nm, which are indexed to the (140) and (120) plane of Bi_19_Br_3_S_27_, respectively. After Ni incorporation, an overlayer emerged on the BBS nanoneedles, exhibiting a thickness that scaled with the Ni content (Figure 1c and Figure S2). In particular, the high‐resolution TEM (HRTEM) image of BBSN‐8 (Ni/Bi = 8:100) in Figure 1d,e revealed a distinct crystalline‐amorphous boundary, indicating the presence of an amorphous overlayer on the surface of BBS. To further identify the structure of crystalline/amorphous structure, the HRTEM characterization and the corresponding elemental mapping tests were conducted. As shown in Figure 1f, the Bi and Br elements are evenly dispersed throughout the whole nanoneedle tips, whereas the Ni element predominantly concentrated in the amorphous shell and S is uniformly distributed throughout the entire region. Furthermore, the energy‐dispersive spectroscopy (EDS) line scanning profiles in Figure 1g showed that a gradual outward increase in Ni concentration. These results suggest that the outer amorphous layer consists of NiS*_x_*, which is in situ formed on the BBS surface. The diffraction spots and diffraction rings in the corresponding selected‐area fast fourier transform (FFT) patterns in Figure S3 further verifies the crystalline structure of BBS and the amorphous phase of NiS*_x_*.

**FIGURE 1:**
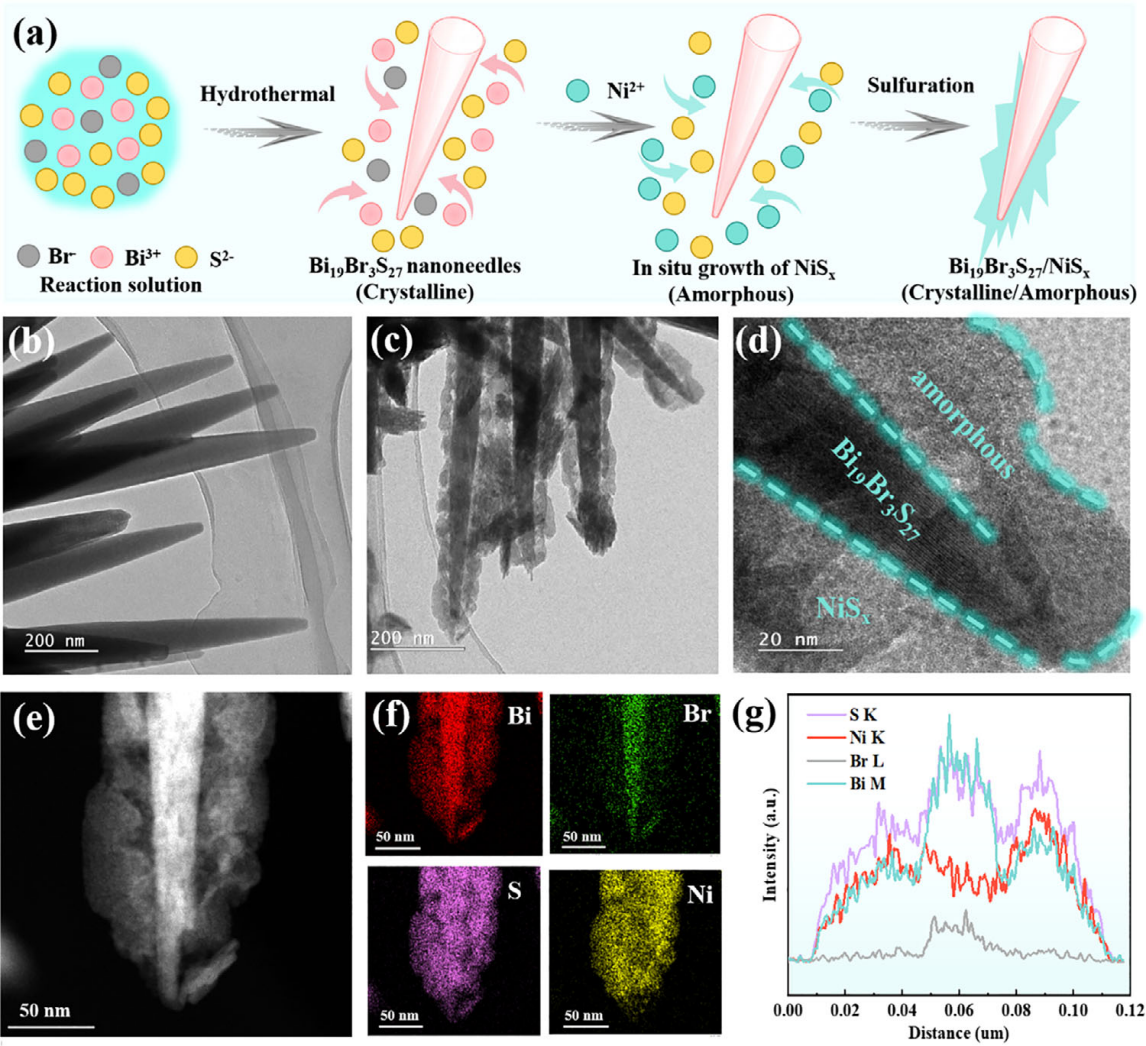
(a) Schematic drawing of the synthesis route of Bi_19_Br_3_S_27_/NiS*_x_*. The TEM images of (b) BBS and (c) BBSN‐8. (d) HRTEM, (e) HAADF‐STEM, (f) corresponding elemental mappings, and (g) EDS line scanning profiles of BBSN‐8.

The phase structure of BBSN was analyzed by x‐ray diffraction (XRD) and Raman characterizations. As illustrated in Figure 2a, all samples exhibit characteristic diffraction patterns corresponding to the hexagonal phase of Bi_19_Br_3_S_27_ (JCPDS No. 70–0202), which verifies that BBS possesses high crystallinity. For the BBSN‐*x* with different molar ratios of Ni/Bi, there were no new diffraction peaks emerged in the XRD patterns, but the peak intensity of BBS decreased slightly. This may be owing to the excess sulfur species released from thiourea during the synthesis process, which could readily react with Ni^2+^, resulting in the preferential formation of NiS*_x_* under sulfur‐rich conditions. The Raman spectra were further used to detect the crystallinity of BBSN. Compared to BBS, the Raman spectrum of BBSN‐8 with amorphous NiS*_x_* exhibits a pronounced peak broadening at 264 cm^−1^ (Figure 2b), suggesting that the interaction of BBS with the introduction of Ni, which is in favor of surface reconstruction of BBS [25].

**FIGURE 2:**
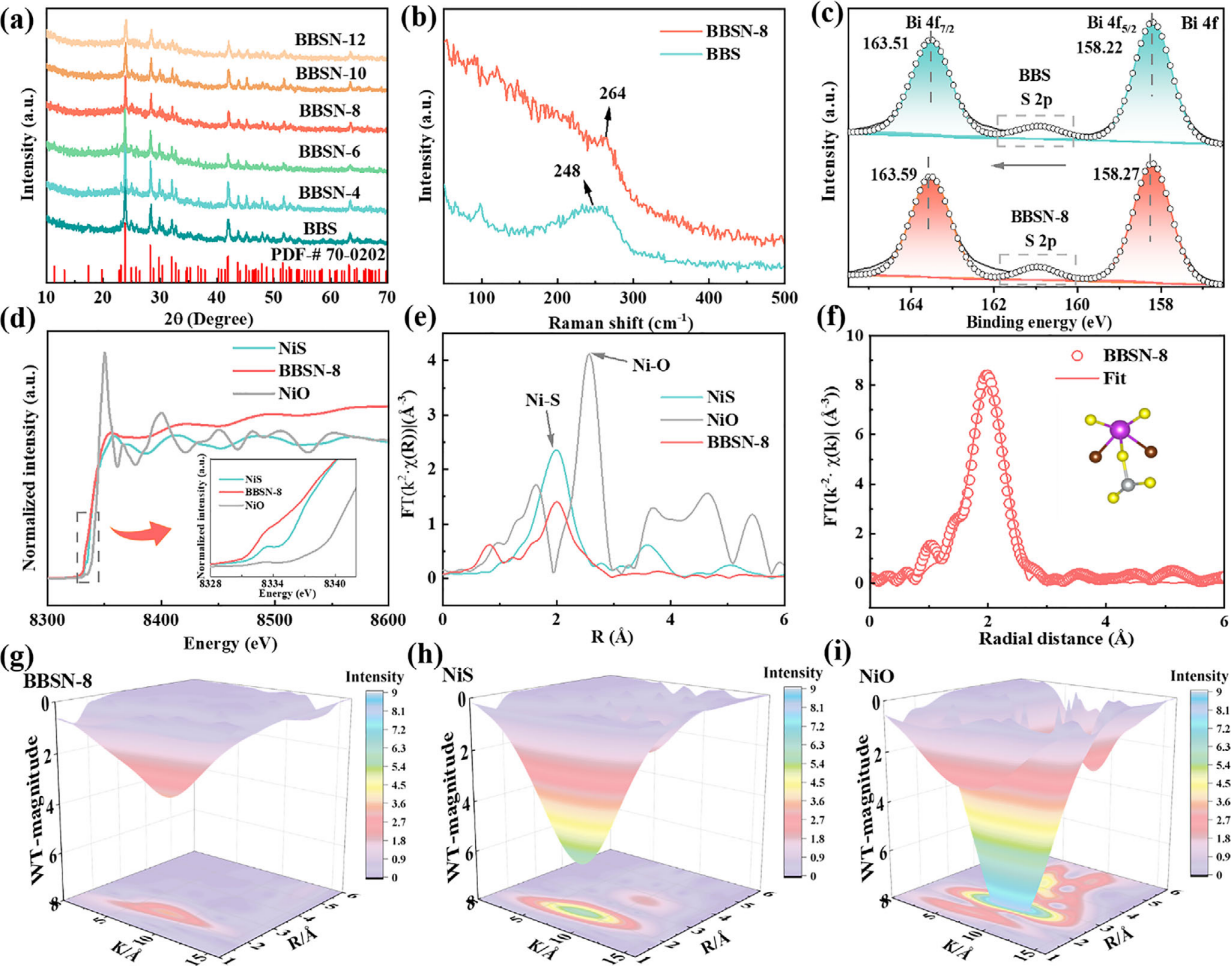
(a) The XRD patterns of BBSN. (b) Raman spectra, (c) Bi 4f XPS spectra of BBS and BBSN‐8. (d) XANES and (e) FT‐EXAFS of Ni K‐edge spectra for NiO, NiS, and BBSN‐8. (f) EXAFS fitting results of Ni K‐edge at k‐space of BBSN‐8. 3D WT‐EXAFS at Ni K‐edge for (g) BBSN‐8, (h) NiS, and (i) NiO.

The surface chemical state evolution from BBS to BBSN‐8 transformation was investigated through x‐ray photoelectron spectroscopy (XPS) tests. The Bi 4f XPS spectra of BBS and BBSN‐8 are shown in Figures 2c and S4. Two characteristic peaks emerge at the binding energies of 163.59 eV and 158.27 eV, corresponding to the Bi 4f_7/2_ and Bi 4f_5/2_, respectively [26]. After loading of NiS*_x_*, the Bi 4f in BBSN‐8 shifts 0.06 eV toward high binding energy compared to BBS, indicating that the electron cloud density around Bi decreased after deposition of NiS*_x_*. This may be attributed to interfacial charge redistribution between BBS and NiS_x_, wherein the sharing of S^2^^−^ drives the establishment of interfacial coupling that accelerates charge migration.

To probe the atomic coordination structure of BBSN‐8, x‐ray absorption near‐edge structure spectra (XANES) measurements were carried out at the Ni K‐edge. As shown in XANES spectra (Figure 2d), the absorption edge position of BBSN‐8 closely overlaps with that of NiS, indicating that Ni predominantly exists in the +2 valence state. The Fourier transform extended x‐ray absorption fine structure (FT‐EXAFS) spectra in Figure 2e,f display an obvious peak at approximately 2.02 Å, corresponding to the first‐shell Ni─S coordination, consistent with the Ni─S bond in NiS, confirming the formation of Ni─S bonds in BBSN‐8 [25]. Notably, the weakened EXAFS intensity compared to NiS references implies a higher degree of disorder local structural around the Ni sites in BBSN‐8. To gain deeper insight into the coordination environment, 3D wavelet transform‐EXAFS (3D WT‐EXAFS) analysis was further employed. As illustrated in Figure 2g–i, the 3D WT contour map of BBSN‐8 exhibits a characteristic intensity feature located at ∼2.1 Å along the Y radial distance axis, which matches well with the Ni─S scattering signal observed in NiS, while differing from that of NiO, thereby excluding significant Ni─O coordination. Quantitative EXAFS fitting results (Figures S5 and S6 and Table S2) confirm that Ni atoms are mainly coordinated with S atoms. Importantly, the coordination number of the Ni─S shell is significantly reduced (2.5) compared to crystalline NiS (CN = 6), indicating a highly unsaturated coordination environment [27]. Meanwhile, the negative ΔR values and relatively large Debye−Waller factors further confirm bond contraction and substantial structural disorder. Collectively, these results demonstrate that Ni species in BBSN‐8 predominantly exist in a low‐coordinated and structurally disordered Ni─S configuration.

### 2.2 Photothermal Catalytic CO_2_ Reduction

The photothermal catalytic performances of BBS and BBSN were evaluated under full‐spectrum light irradiation (Figure S7). Product analysis by gas chromatography and nuclear magnetic resonance (^1^H NMR spectroscopy) (Figure S8) tests reveal that C_2_H_4_ and CO are the primary carbon‐containing products, while only trace amounts of H_2_ are detected. Furthermore, a volcano‐type trend of C_2_H_4_ yield and selectivity on BBS with different Ni loading is observed in Figures S9 and 3a. Under full‐spectrum light illumination, the BBSN‐8 catalyst achieves the highest activity (41.38 µmol g^−1^ h^−1^) with a remarkable selectivity of 96.8%, representing an 8.8‐fold increase compared to BBS (4.66 µmol g^−1^ h^−1^, 81.5%). This significantly enhanced yield highlights the crucial role of NiS*_x_* in promoting the rapid coupling of C_1_ intermediates toward C_2_H_4_. To further assess the light‐to‐heat conversion ability, catalytic performance for BBSN‐8 was evaluated under different irradiation intensities. As shown in Figure 3b, C_2_H_4_ yields increase substantially with increasing light intensity from 0.2 to 0.68 W cm^−2^. Notably, under concentrated solar irradiation (0.68 W cm^−2^), BBSN‐8 reaches an activity of 43.38 µmol g^−1^ h^−1^ with selectivity of 96.8%, which is 3.6 times higher than that of under non‐concentrated illumination (11.92 µmol g^−1^ h^−1^, 0.2 W cm^−2^), underscoring the excellent photo‐thermal conversion ability of BBSN‐8 [28, 29]. Meanwhile, the results of surface temperature under different irradiation times showed that BBSN‐8 reaches a notably higher steady‐state temperature (240°C) than BBS (200°C) (Figure S10). This result further demonstrated that NiS*_x_* incorporation significantly enhances light‐to‐heat conversion efficiency, which is closely associated with catalytic performance.

**FIGURE 3:**
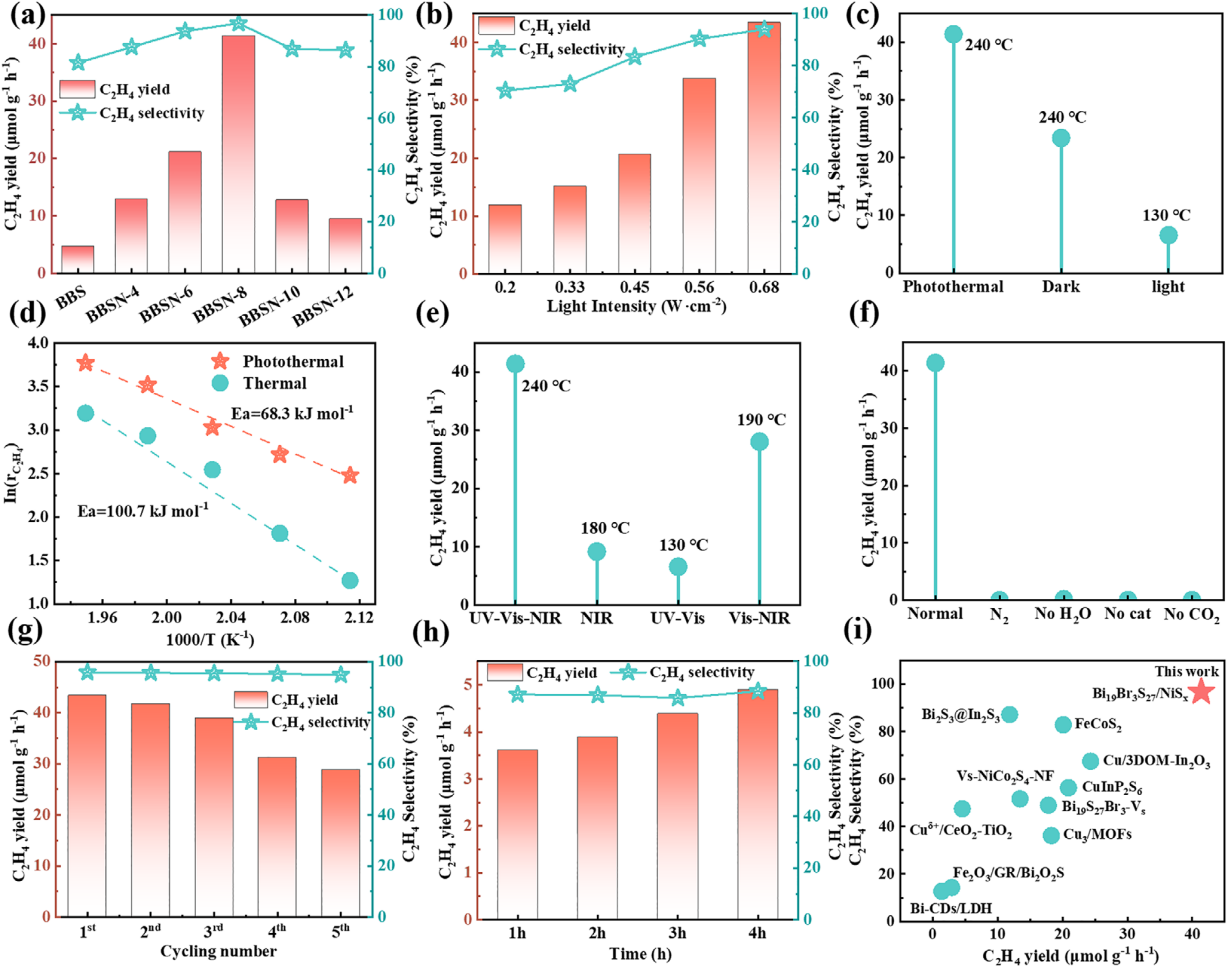
(a) C_2_H_4_ yields and selectivity of CO_2_ reduction on BBS and BBSN under full‐spectrum light irradiation. C_2_H_4_ yields and selectivity of CO_2_ reduction on BBSN‐8 at different (b) irradiation intensities and (c) reaction system. (d) Apparent activation energy of BBSN‐8 under photothermal conditions and in dark. C_2_H_4_ yields on BBSN‐8 under different (e) light source irradiation and (f) reaction atmospheres. (g) Cycling tests of BBSN‐8 for CO_2_ reduction. (h) C_2_H_4_ yields and selectivity of BBSN‐8 under atmospheric pressure conditions. (i) Comparison of the performance of BBSN‐8 with reported catalysts in C_2_H_4_ production from CO_2_.

To further disentangle the contributions of thermal effects and photocatalytic effects over the BBSN‐8, the CO_2_ reduction experiments were conducted under distinct reaction environments. In dark at 240°C, BBSN exhibits a C_2_H_4_ yield of 23.39 µmol g^−1^ h^−1^ that accounts for 53.8% of C_2_H_4_ yield under full‐spectrum light irradiation, indicating that thermal plays an important role in the C_2_H_4_ generation (Figure 3c). Besides, the apparent activation energy for C_2_H_4_ production decreases from 100.7 kJ mol^−1^ under thermal catalysis to 68.3 kJ mol^−1^ under full‐spectrum light illumination (Figure 3d), indicating that the reaction kinetics are enhanced through the synergistic effect between photocatalytic and thermocatalytic processes. To further explore the underlying role of light, CO_2_ reduction tests were subsequently carried out under different light sources. As shown in Figure 3e, the surface temperature of BBSN‐8 under visible‐near (Vis−NIR) light (190°C) is similar with that achieved under NIR light (180°C), while the C_2_H_4_ yield under Vis−NIR irradiation (28.1 µmol g^−1^ h^−1^) is markedly higher than under NIR excitation (9.13 µmol g^−1^ h^−1^), revealing that temperature rise induced by photothermal effect is not the sole driving force, and photoexcited charge carriers generated under light irradiation play a critical role in enabling C─C coupling toward C_2_H_4_. Combining these results, it could be concluded that the photothermal CO_2_ conversion in BBSN is predominantly initiated by a synergistic process for photocatalytic and thermal catalytic reactions.

To identify the source of CO_2_ reduction products over the BBSN‐8 catalyst, a series of control experiments were performed. As illustrated in Figure 3f, no carbon‐containing products were observed under N_2_ atmosphere or in the absence of CO_2_ and catalyst. The ^13^CO_2_ labeling experiment in Figure S11 further confirmed that the generated C_2_H_4_ originates exclusively from CO_2_ reduction, rather than from any extraneous sources (The detected CO_2_ signals arise from the filling of natural CO_2_ into the reactor). The stability of the BBSN‐8 catalyst is examined by cycling tests under full‐spectrum illumination (Figure 3g). The C_2_H_4_ selectivity of the BBSN‐8 is well maintained with slightly decreased yield in the CO_2_ reduction reaction after five cycles. Besides, XRD and XPS analyses in Figure S12 before and after reaction confirm the good structural and chemical stability of BBSN‐8. Moreover, the photothermal catalytic atmospheric CO_2_ performance of the BBSN‐8 is evaluated and shown in Figure 3h. Even under low CO_2_ concentration conditions, BBSN‐8 still has a good CO_2_ reduction performance, in which the yield and selectivity of C_2_H_4_ could reach 4.9 µmol g^−1^ h^−1^ and 88.4%, respectively. In addition, it could be seen that BBSN‐8 has a high selectivity for C_2_H_4_ than most reported catalysts (Figure 3i and Table S3). The results demonstrate that the BBSN‐8 catalyst shows excellent photothermal catalytic CO_2_ reduction with excellent C_2_H_4_ selectivity_._

### 2.3 Charge Carrier Separation Kinetics

The interfacial electron migration behavior in BBSN was further analyzed by combining XPS analysis with density functional theory (DFT) calculations. As displayed in Figure 4a, the Ni 2p XPS spectrum of BBSN‐8 shows peaks at 853.7 and 871.4 eV corresponding to the Ni^2+^, and peaks at 855.6 eV be assigned to the Ni^3+^ [30]. Under light irradiation, the binding energies of Ni in BBSN‐8 shift toward lower values, whereas the of Bi shift toward higher energies (Figure 4b). This opposite shift indicates that photogenerated electrons migrate from BBS toward NiS*_x_*, suggesting that NiS*_x_* serve as co‐catalyst and promotes interfacial electronic coupling [31]. To investigated the directional charge transfer, the work functions of BBS and NiS*_x_* were calculated to be 5.036 and 5.257 eV, respectively (Figure 4c,d). Upon contact, the difference in Fermi levels (*E*_f_) induces the migration of electrons from BBS to NiS*_x_*, establishing an internal electric field (Figure 4e). Charge density difference calculations further confirm this process, where obvious electron accumulation occurs around the Ni sites in NiS*_x_* region (Figure S13). Moreover, the 2D charge density difference plot in Figure 4f reveals a clear charge redistribution in BBSN. In BBS, the charge density is primarily confined around individual atomic sites, indicating limited electronic interaction between neighboring atoms. In contrast, the introduction of Ni induces distinct electron accumulation (green color) and depletion (blue color) regions around Ni sites. This spatial separation of charge suggests the formation of local dipoles and an internal electric field at the interface [32]. Furthermore, Bader charge analysis in Figure S14 was employed to quantitatively evaluate electron redistribution among Ni, S, and Bi atoms in BBSN. The results indicate that the Ni atom gains on average ∼0.12 |e| from S, while each S atom accepts ∼0.04 |e| from Bi within the Ni─S─Bi of BBSN, confirming that electrons are transferred from BBS to NiS*_x_*. Additionally, DFT calculations further demonstrate that the distance between Bi and Ni decreases from 4.02 Å to 3.64 Å after interface formation (Figure S15), indicating that the formed Ni─S─Bi linkage effectively bridge Bi and Ni sites. The unique Ni─S─Bi bridge could induce strong orbital hybridization and establish spatially coupled dual‐metal active sites capable of stabilizing reaction intermediates [1]. Based on these results, bandgap structure diagram and charge transfer mechanism for BBSN under light irradiation were proposed (Figure S16). Specifically, the built‐in electric field accelerates separation and directional migration of photogenerated electrons from BBS to NiS*_x_*, which favors the C─C coupling reaction.

**FIGURE 4:**
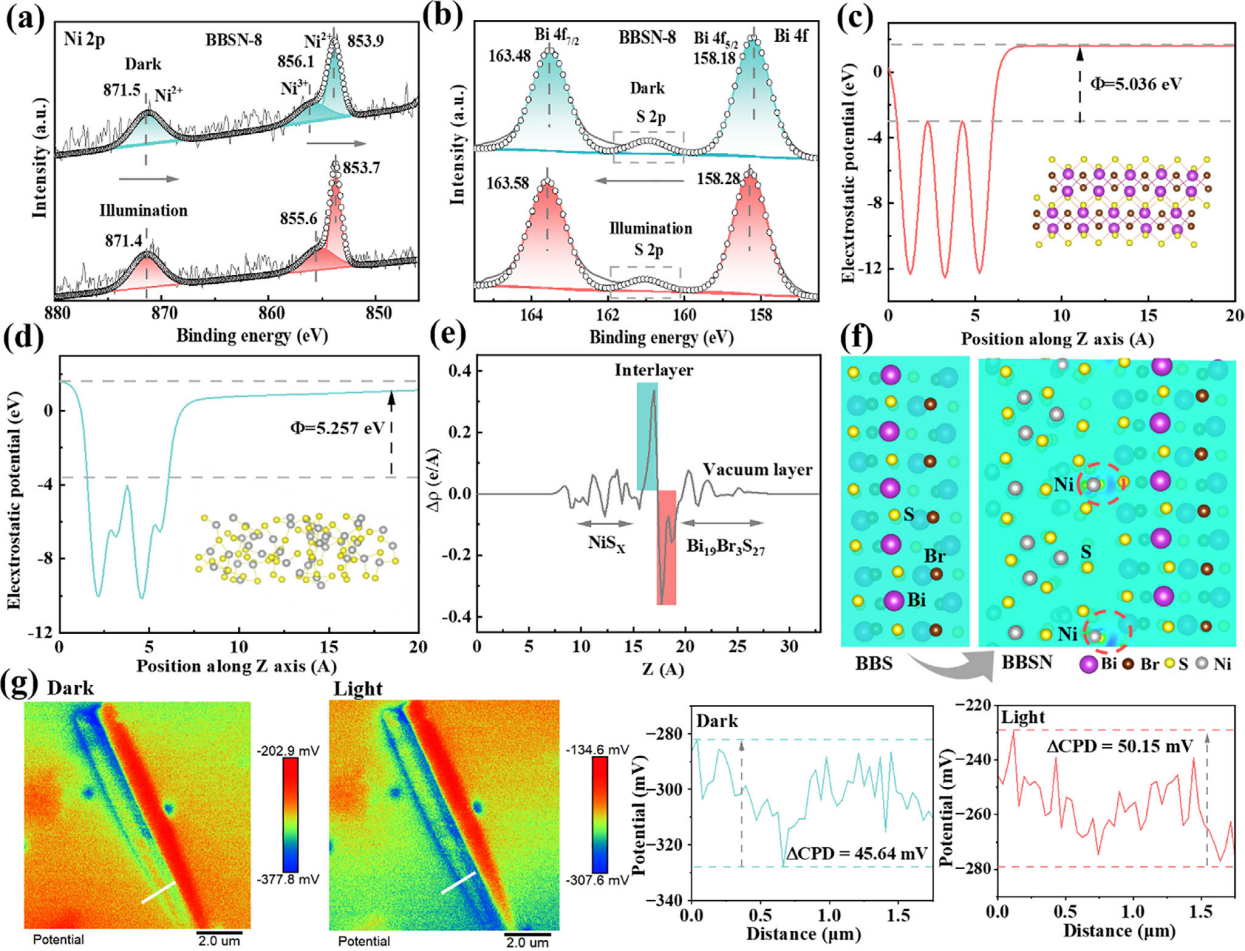
(a) Ni 2p, (b) Bi 4f XPS spectra of BBSN‐8 in dark and under light illumination. Electrostatic potentials of (c) BBS and (d) NiS*_x_*. (e) The corresponding planar average differential charge in BBSN. (f) 2D charge density difference plot of BBSN. (g) Kelvin probe force microscopy (KPFM) surface potential maps and the corresponding line profiles of BBSN‐8 in dark and under light irradiation.

The optical and electronic properties of BBSN were further examined. UV−Vis−NIR spectra in Figure S17 show that BBSN possesses stronger light absorption intensity than BBS across the entire spectral region. Meanwhile, the bandgap narrows from 1.55 eV for BBS to 1.48 eV for BBSN‐8 (Figure S18), favoring NIR absorption and photothermal conversion. Furthermore, the results of Mott−Schottky tests show that the flat‐band potential of BBSN‐8 is −0.72 V vs. Ag/AgCl electrode, which could be estimated to be approximately −0.62 V due to *n*‐type semiconductors (Figure S19) [33]. This is more negative than that of BBS (−0.49 V), which provides stronger reduction ability for CO_2_ reduction to C_2_H_4_ (E°(CO_2_/C_2_H_4_) = −0.34 V vs. NHE, pH 7) (Figure S20). Photoelectrochemical measurements further verify the enhanced carrier dynamics in BBSN. Compared with BBS, BBSN‐8 displays a smaller arc diameter than that of BBS (Figure S21), indicating more efficient charge transfer and suppressed carrier recombination. The role of thermal excitation during the photothermal catalytic process was further clarified by temperature‐dependent photoluminescence (TD‐PL) spectra (Figure S22). A progressive decrease of PL intensity with rising temperature was observed, indicating that thermal energy promotes the separation of photogenerated carriers within BBSN [34]. In addition, the change in contact potential difference (ΔCPD) value of BBSN‐8 increases from 45.64 mV in dark to 50.15 mV under illumination (Figure 4g), indicating an enhanced interfacial potential difference and more pronounced directional charge migration from BBS to NiS*_x_* under light irradiation [35]. Additionally, surface charge properties were evaluated by zeta potential measurements (Figure S23). Introduction of NiS*_x_* reversed the zeta potential of BBS from +5.62 mV to −0.22 mV, signifying an electron‐rich surface state environment favorable for the adsorption and activation of CO_2_ molecules, thereby contributing to improved catalytic activity.

### 2.4 Theoretical Analysis of Orbital Hybridization

DFT theoretical calculations were carried out to elucidate the cooperative electronic interactions within the Ni─S─Bi structure of BBSN‐8. The crystal orbital Hamilton population (COHP) analysis provides direct insight into the enhanced C_2_H_4_ production upon Ni incorporation. In BBS, the adsorption is dominated by an excessively strong Bi─C interaction (−3.43 eV), which may lead to over‐stabilization of intermediates and hinder their subsequent coupling (Figure 5a) [36, 37]. After introducing Ni, the Bi─C interaction is significantly weakened (−2.00 eV), while additional Ni−C interactions (−1.10 eV) are established, indicating a redistribution of carbon binding sites (Figure 5b). This moderated and diversified carbon‐metal interaction is critical for preventing site poisoning and enabling sufficient mobility of intermediates [38]. Meanwhile, the enhanced Bi─O (−1.99 eV), Ni─O (−0.73 eV), and S─O (−0.51 eV) interactions suggest a strengthened stabilization of oxygen‐containing intermediates, which facilitates maintain adsorption balance during reaction (Figure 5c,d). More importantly, this cooperative adsorption enables synergistic stabilization of both the electrophilic carbon and nucleophilic oxygen termini, thereby inducing pronounced molecular bending and facilitating π* orbital activation of CO_2_. Meanwhile, this process facilitates carbon‐containing intermediates coverage and activation, thereby promoting C─C coupling and leading to the significantly increased C_2_H_4_ production.

**FIGURE 5:**
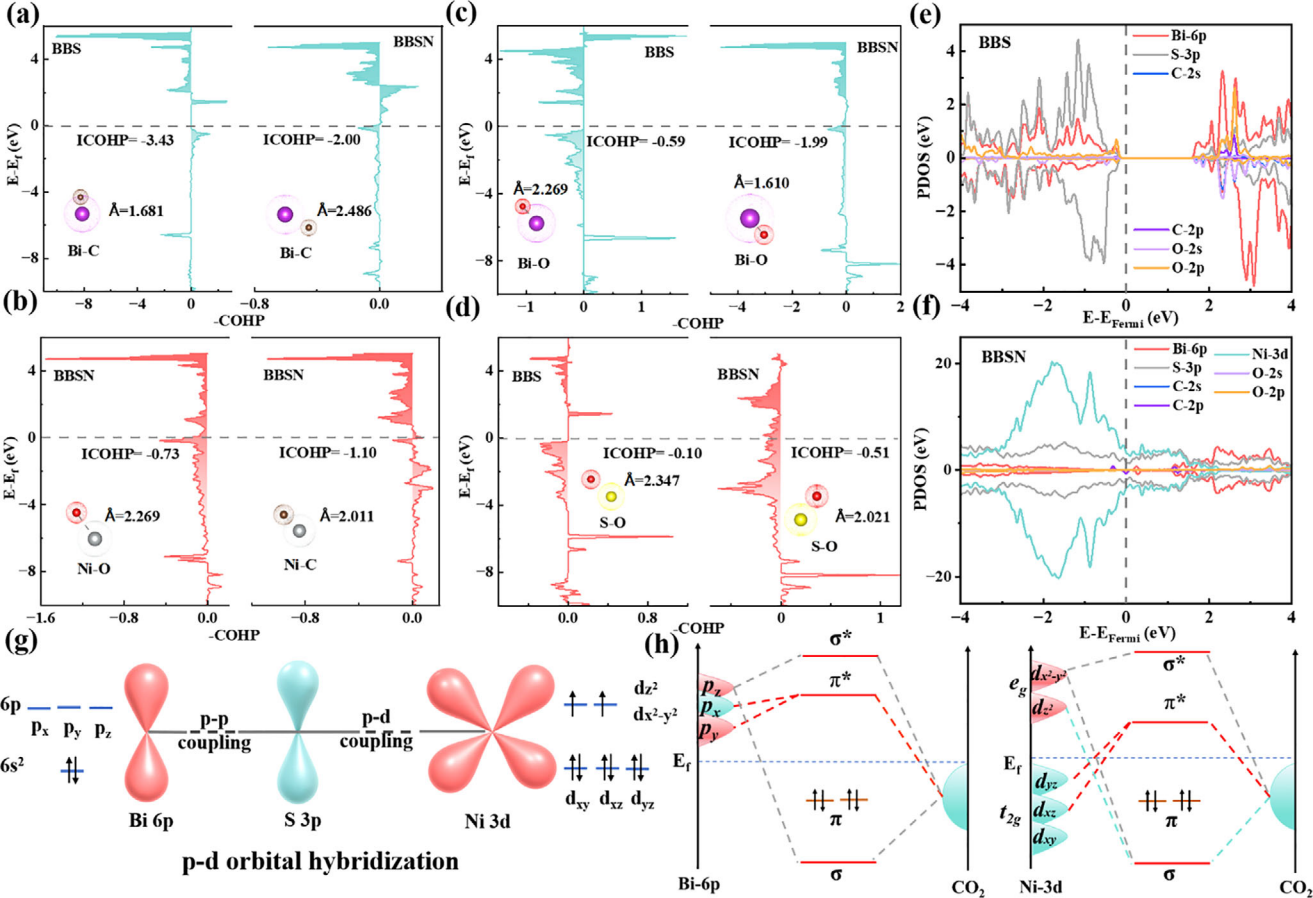
COHP analysis of (a) Bi─C bond, (b) Ni─O and Ni─C, (c) Bi─O, (d) S─O of BBS and BBSN‐8 react with CO_2_, respectively. DOS analysis showing CO_2_ interaction with (e) BBS and (f) BBSN, respectively. (g) Schematic depiction of p‐d orbital hybridization for BBSN. (h) Schematic energy‐level diagram illustrating the orbital interactions between Bi/Ni states and CO_2_ molecular orbitals in BBSN.

The density of states (DOS) further clarifies the electronic structure reconstruction of BBS induced by Ni incorporation. For BBS in Figure 5e, the valence band is mainly composed of Bi 6p and S 3p states, while the conduction band is predominantly contributed by Bi 6p states, indicating a relatively localized electronic structure with limited orbital overlap near the *E*_f_. The C 2p states originating from adsorbed CO_2_ are weakly distributed, suggesting localized electronic coupling and insufficient activation ability [39]. After Ni incorporation (Figure 5f), a pronounced Ni 3d state emerges near the *E*_f_, spanning approximately from −2 to 0 eV, which significantly alters the interfacial electronic structure [40]. Notably, the Ni 3d orbitals exhibit strong hybridization with Bi 6p and S 3p states in this energy region, forming a continuous electronic distribution across the interface. More importantly, a clear overlap between Ni 3d, Bi 6p, and the C 2p states is observed near the *E*_f_, implying strengthened orbital interaction between the catalyst surface and CO_2_ [41, 42]. Compared with the results of DOS for BBS, the C 2p states in reaction of BBSN with CO_2_ become more broadened and shift closer to the *E*_f_, suggesting enhanced electronic coupling and improved ability for charge transfer from BBSN into CO_2_ antibonding orbitals, which is consistent with the COHP results, where the adsorption configuration evolves from a single‐site strong Bi─C interaction to a multi‐site cooperative binding mode involving Bi─C, Bi─O, S─O, and Ni─C, which is beneficial for CO_2_ activation and the enhanced C─C coupling performance.

As shown in Figure 5g, the S 3p orbitals act as an electronic bridge, simultaneously interacting with Bi 6p and Ni 3d states to form p‐p and p‐d hybridizations, respectively. Specifically, electron donation from S to the empty Bi 6p orbitals establishes a donor–acceptor p‐p interaction, rendering the Bi sites electron‐deficient. Meanwhile, the strong p‐d hybridization between S 3p and Ni 3d orbitals proceeds via dual channels, involving π‐type coupling with t_2g_ orbitals and σ‐type interaction with e_g_ orbitals, thereby constructing a continuous electron transport network and enabling cross‐interface charge delocalization [18, 43, 44]. Furthermore, as shown in Figure 5h, such electronic structure reconstruction markedly alters the adsorption and activation behavior of CO_2_. In contrast to Bi sites, which interact with CO_2_ primarily through weak p‐π* coupling leading to limited polarization, Ni sites provide both σ donation and π back‐donation pathways. In particular, electron back‐donation from Ni t_2g_ orbitals to the π* antibonding orbitals of CO_2_ effectively weakens the C═O bonds, thereby facilitating CO_2_ activation.

### 2.5 Investigation of C─C Coupling Mechanisms

To explore the greatly improved photothermal catalytic activity of CO_2_ reduction with BBSN‐8, comprehensive structural and electronic characterizations were conducted. The Brunauer–Emmett–Teller (BET) surface area of BBSN‐8 was measured to be 65.026 m^2^ g^−1^, which was larger than that of BBS (18.63 m^2^ g^−1^), thereby providing more accessible sites for CO_2_ adsorption (Figure S24 and Table S4). CO_2_ temperature‐programmed desorption (CO_2_‐TPD) tests in Figure 6a were directly used to reflect the adsorption ability of BBSN‐8 and BBS for CO_2_. Compared with BBS, BBSN‐8 displays a pronounced desorption peak in the range of 200 to 400°C, indicating that BBSN‐8 has stronger affinity for CO_2_ compared to BBS [45]. DFT calculations were further performed to investigate the adsorption behavior of CO_2_ on BBS and BBSN‐8. The optimized adsorption configurations for BBS and BBSN reveal that the C in CO_2_ is adsorbed on Bi and/or Ni, indicating that the C in CO_2_ is more easily adsorbed on the surface of Bi or Ni. The adsorption energy of CO_2_ on BBS is calculated to be −0.36 eV (Figure 6b), suggesting a relatively weak interaction with BBS. After the incorporation of NiS_x_, the adsorption energy at the Bi sites of BBSN‐8 decreases significantly to −1.452 eV. More importantly, when CO_2_ is located at the interface between Bi and Ni sites, the adsorption energy further decreases to −2.185 eV, demonstrating that the Bi─Ni dual sites constitute the most favorable adsorption configuration with strong synergistic interaction. The Bader charges show that the charge transferred from BBSN and BBS to CO_2_ is 0.48 e^−^, 0.17 e^−^, respectively (Figure S25), indicating that the Ni 3d orbitals strongly hybridize with the C 2p of CO_2_, enabling effective electron back‐donation.

**FIGURE 6:**
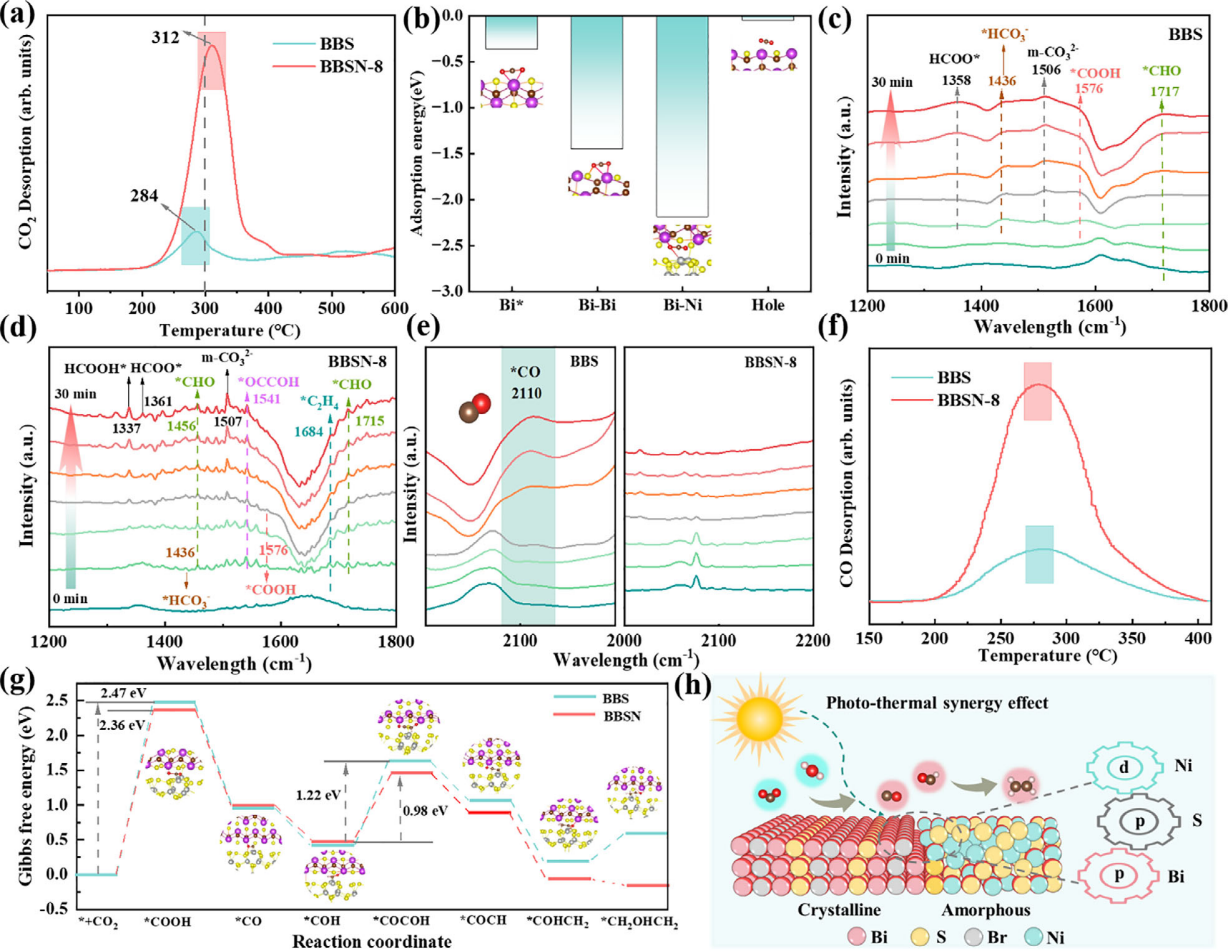
(a) CO_2_‐TPD curve of BBS and BBSN‐8. (b) Adsorption energy of CO_2_ at different adsorption sites in BBS and BBSN. In situ DRIFTS for photothermal catalytic CO_2_ reduction of (c) BBS and (d) BBSN‐8 under full‐spectrum irradiation. (e) Enlarged in situ DRIFTS of BBS and BBSN‐8 under full‐spectrum irradiation. (f) CO‐TPD curve of BBS and BBSN‐8. (g) Gibbs free energy diagrams of CO_2_ reduction process over the BBS and BBSN. (h) The schematic diagram of photothermal catalytic CO_2_ reduction into C_2_H_4_ for BBSN.

In situ diffuse reflectance infrared Fourier transform spectroscopy (in situ DRIFTS) tests were employed to investigate the reaction intermediates in the photothermal catalytic CO_2_ reduction. As shown in Figure 6c, several new peaks emerged in the in situ DRIFTS spectra of BBS during the illumination period from 0 to 30 min, indicating the generation of several different reaction intermediates. The presence of peaks at 1576 cm^−1^ corresponded to the COOH* intermediates, which was a key intermediate for the conversion of CO_2_ to C_1_ and other fuels [46]. Moreover, the peak observed at 1358, 1436, and 1506 cm^−1^ are identified as originating from the HCOO*, *HCO_3_, and m‐CO_3_^2−^ groups, respectively [33, 47]. Similarly, the characteristic peak at 1717 cm^−1^ was attributed to the presence of the CHO* groups [48]. In contrast, new characteristic peaks appeared in the in situ DRIFTS spectra of BBSN‐8 under the same conditions (Figure 6d). The peak at 1541 cm^−1^ is associated with *OCCOH [1], which is recognized as a key intermediate for C─C coupling and C_2_H_4_ generation. Meanwhile, the peak at 1684 cm^−1^ was ascribed to C_2_H_4_* stretching vibration of C_2_H_4_, confirming the formation of C_2_H_4_ [49, 50]. Notably, no detectable OCCOH* signal is observed on BBS, suggesting that the OCCOH* group may be a prominent intermediate in the CO_2_ reduction reaction pathway facilitated by the BBSN. Besides, obvious *CO accumulation (2110 cm^−1^) is detected on BBS, whereas the *CO signal on BBSN‐8 becomes much weaker (Figure 6e), which may be due to the rapid conversion of *CO intermediates after Ni incorporation [51, 52]. To clarify this difference, CO adsorption behavior was further examined by CO‐TPD and DFT calculations. As shown in Figure S26, the CO adsorption energy (*E*_ads_) in BBSN (−1.628 eV) is much higher than that in BBS (−0.341 eV), suggesting that p‐d orbital hybridization significantly strengthens CO adsorption stability. Meanwhile, the results of CO‐TPD in Figure 6f show that BBSN‐8 has a larger peak area around 200–400°C compared to BBS, further confirming that BBSN‐8 has stronger CO adsorption ability. Therefore, the absence of detectable *CO in the in situ DRIFTS spectra of BBSN‐8 may be attributed to the rapid conversion of *CO intermediates on the surface.

To gain deeper insight into the reaction energetics, Gibbs free energy calculation was further carried out. As shown in Figure 6g, the conversion of adsorbed CO_2_ to the *COOH intermediate involves a pronounced uphill free energy of approximately 2.47 eV for BBS, identifying this step as the potential‐determining step. Moreover, the subsequent generation of *OCCOH intermediate by C─C coupling reaction between *CO and CHO*, requires an additional energy input of about 1.22 eV, indicating that the formation of C_2_ intermediates is more thermodynamically unfavorable on the surface of BBS [53]. In contrast, BBSN exhibits a slightly reduced free energy barrier of approximately 2.36 eV for the formation of the COOH* intermediate, suggesting a modest enhancement in CO_2_ activation. More importantly, the free energy requirement for the formation of the *OCCOH intermediate is significantly decreased to around 0.98 eV, representing a substantial reduction compared to BBS, which greatly facilitates the C─C coupling process [54]. Furthermore, the subsequent intermediates along the C_2_ pathway, such as *COCH and *COHCH_2_ species, display more negative ΔG values on BBSN, indicating that the surface of BBSN is more conducive to C─C coupling reaction. As illustrated in Figure 6h, under photo‐thermal synergy, newly formed p‐d orbital coupling at the BBSN interface promotes charge redistribution and selective C_2_H_4_ formation via facilitated C─C coupling.

## 3 Conclusion

In summary, an in situ constructed crystalline/amorphous Bi_19_Br_3_S_27_/NiS*_x_* heterostructure was developed for highly selective photothermal CO_2_ conversion toward C_2_H_4_. The optimized BBSN‐8 catalyst achieved a C_2_H_4_ production rate of 41.38 µmol g^−1^ h^−1^ with a selectivity of 96.8%, representing an 8.8‐fold enhancement over pristine Bi_19_Br_3_S_27_. Mechanistic investigations reveal that the in situ generated NiS*_x_* induces strong p‐d orbital hybridization and transforms CO_2_ activation from localized Bi─C single‐site adsorption into a cooperative multi‐site configuration involving Bi─C, Bi─O, S─O, and Ni─C interactions. Consequently, the C─C coupling barrier is significantly reduced, accompanied by the emergence of *OCCOH intermediates and suppressed *CO accumulation, thereby promoting selective C_2_H_4_ formation. This work highlights orbital‐coupling‐driven pathway regulation as an efficient strategy for selective multi‐carbon photothermal catalytic CO_2_ conversions.

## Author Contributions

**Guangmei Gan**: software, data curation, formal analysis, validation, writing ‐ original draft. **Zhixiong Yang**: formal analysis, data curation, writing – review and editing. **Mengying Wang**: data curation, validation. **Yang Shi**: writing – review and editing, formal analysis, validation. **Yuan Li**: writing – review and editing, validation. **Gaoke Zhang**: conceptualization, investigation, funding acquisition, writing – review and editing, methodology, project administration, supervision, resources. **Jie Wu**: funding acquisition, writing – review and editing, supervision.

## Conflicts of Interest

The authors declare no conflicts of interest.

## Supporting information




**Supporting File**: anie74821‐sup‐0001‐SuppMat.docx.

## Data Availability

The data that supports the findings of this study are available in the Supporting Information of this article.
