# Supplementary material for: Constructing p‐d Orbital Hybridization via In Situ Generation of Bi19Br3S27/NiS x for Highly Selective Photothermal CO2 Reduction to C2H4
Source: Angew Chem Int Ed Engl. 2026 Sep 15;65(45):e1626004. doi: 10.1002/anie.1626004 (PMC13725584; doi:10.1002/anie.1626004)
Supplement: Supplementary file 1 [file ANIE-65-e1626004-s001.docx]

**Supplementary Information**

**Constructing p-d Orbital Hybridization via In situ Generation of Bi_19_Br_3_S_27_/NiS*ₓ* for Highly Selective Photothermal CO_2_ Reduction to C_2_H_4_**

Guangmei Gan^a^, Zhixiong Yang^a^, Mengying Wang^a^, Yang Shi^b^, Yuan Li^a^, Gaoke Zhang*^a^ and Jie Wu*^b^

^a^ *Hubei Key Laboratory of Mineral Resources Processing and Environment, Key Laboratory of Green Utilization of Critical Non-metallic Mineral Resources, Ministry of Education, State Key Laboratory of Silicate Materials for Architectures, Wuhan University of Technology, 122 Luoshi Road, Wuhan 430070, People’s Republic of China*

^b^ *Department of Chemistry, National University of Singapore, 3 Science Drive 3, Singapore 117543, Singapore*

**Corresponding author: Gaoke Zhang, Jie Wu*

*E-mail address: gkzhang@whut.edu.cn, chmjie@nus.edu.sg*

**Experimental Details**

**Preparation of** **Bi_19_Br_3_S_27_/NiS*_x_***

The Bi_19_Br_3_S_27_/NiS*_x_* were obtained through a modified hydrothermal strategy adapted from earlier reports [1]. In a typical synthesis, 4 mmol of thiourea (0.30 g), 3.2 mmol of KBr (0.38 g), and 1.2 mmol of Bi(NO_3_)_3_∙5H_2_O (0.58 g), and varying amounts of Ni(NO_3_)_2_∙6H_2_O were dissolved in 80 mL of ethylene glycol under vigorous stirring for 30 min to achieve uniform mixing. The solution was then sealed in a 100 mL Teflon-lined stainless-steel autoclave and maintained at 130°C for 12 h. After cooling to room temperature, the black solids were collected by centrifugation, repeatedly washed with deionized water and absolute ethanol, and subsequently dried in a vacuum oven at 60 °C for 24 h. The obtained materials were denoted as BBSN-4, BBSN-6, BBSN-8, BBSN-10, and BBSN-12, according to the Ni/Bi molar ratio (e.g., BBSN-8 corresponds to Ni/Bi = 8:100). For comparison, pristine Bi_19_Br_3_S_27_ was synthesized under identical conditions without introducing Ni(NO_3_)_2_·6H_2_O.

**Catalyst Characterization**

The phase and crystallinity of the samples were determined by X-ray diffraction (XRD) analysis on a D/MAX-RB diffractometer and operated at 40 kV voltage and 50 mA current. The morphology and microstructure of the samples were measured by scanning electron microscopy (SEM, JSM-5610LV), transmission electron microscopy (TEM) using a Fet talos F200X electron microscope, which was operated at an accelerating voltage of 200 KV. Nitrogen adsorption-desorption isotherms of the samples were measured on a Quantachrome autosorb iQ2 adsorption automatic instrument after pretreatment at 150°C under vacuum for 10 h. The UV-Vis diffuse reflectance spectra (DRS) of the as-prepared samples were recorded by a UV-VIS-NIR Spectrophotometer (Shimadzu UV-3600 Plus, Japan). Raman spectra were carried out on an inVia Reflex-Renishaw spectrometer in the anti-Stokes range of 100-1500 cm^-1^. The catalysts were excited using a He-Ge laser with excitation wavelength of 532 nm. X-ray photoelectron spectra (XPS) were measured on a PHI Quantera system with a monochromatic Al Kα source. The elemental compositions of the samples were determined by inductively coupled plasma optical emission spectroscopy (ICP-OES) using an Agilent 5110 instrument. The Kelvin Probe Force Microscopy (KPFM) was carried out on a Bruker Dimension Icon atomic force microscope to confirm the surface potential of the obtained sample under dark and light.

CO_2_ temperature-programmed desorption mass experiments were conducted on the Hiden DECRA, UK. The sample was pretreated with helium gas (30 mL/min) at 200 ℃ for 1 h, then cooled down to 50 ℃ at the same flowing stream, and the inlet gas was switched to 10% CO_2_/He gas 1 h until the catalyst was saturated with adsorption. Then, the gas was switched to pure He gas to remove excess CO_2_. Finally, in a helium flow, the sample was heated from 50 ℃ to 500 ℃ (at a rate of 10 ℃/min).

CO temperature-programmed desorption (CO-TPD) were conducted on AutoChem II 2920. The sample was pretreated in helium gas (50 mL/min) at 200 ℃ for 1h, after cooled down to 50 ℃, the gas was switched to the 10% CO/Ar for 30 min until the mass spectrometry baseline was stable. Then the samples were heated to 400 ℃ at a rate of 10 ℃/min in a 10% CO/Ar mixed gas flow of 30 mL/min. The CO consumption was detected by TCD and the weight of the samples were used to calibrate the signal.

The electrochemical impedance spectroscopy (EIS) is conducted under open-circuit potential with 0.01 to 1 × 10^3^ Hz frequency range and 0.005 V amplitude. The photocurrent response and Mott-Schottky cure (MOS) of different materials are conducted on CHI-660D electrochemical workstation with a three-electrode system. In the typical three-electrode system, the working electrode is the fluoride tin oxide (FTO) with the as-prepared photocatalyst, the reference electrode is Ag/AgCl, and the counter electrode was platinum wire. Electrochemical measurements are carried out in Na_2_SO_4_ (0.5 M, pH = 6.8) solution. The photocurrent response is tested under 300 Xe lamp. Transient photocurrent measurements with chopped illumination are also conducted under full spectrum light irradiation (the interval is 10 s for light on and off) at a fixed potential of 0.2 V (vs standard calomel electrode).

**Density functional theory calculation**

The crystalline Bi_19_Br_3_S_27_ (BBS) structure was constructed according to JCPDS No. 70-0202. A crystalline BBS/NiS heterostructure was first established based on the optimized BBS model. To simulate the amorphous NiS*_x_* phase, structural disorder was introduced by randomly removing 15 Ni atoms and 15 S atoms from the NiS region while preserving the interfacial contact with the BBS substrate, followed by full geometry optimization. The optimized structure exhibited diversified Ni coordination environments (CN = 1-5). The resulting crystalline BBS/amorphous NiS*_x_* interface model was used for subsequent calculations. Prior to static calculations, all structures were fully optimized to obtain the lowest-energy configurations. Electronic structure calculations were performed using the generalized gradient approximation (GGA) within the Perdew-Burke-Ernzerh of (PBE) functional. A plane-wave cutoff energy of 400 eV was adopted. The convergence criteria were set to 1×10^-5^ eV for total energy and 0.02 eV Å^-1^ for Hellmann–Feynman forces, respectively.

The adsorption energy of CO_2_ ($E_{ads-{CO}_{2}}$) was calculated by equation:

$E_{ads-{CO}_{2}}$ =$E_{slab-{CO}_{2}}$ - $E_{slab}$ -$E_{{CO}_{2}}$

Where $E_{slab-{CO}_{2}}$ is the energy of the slab surface, which covered by the CO_2_ molecule, $E_{slab}$ is the energy of the clean slab surface, and$E_{{CO}_{2}}$ is the energy of the gaseous CO_2_ molecule.

**Photothermal CO_2_ reduction tests**

The BBSN catalysts (50 mg) were ultrasonically dispersed in 1 mL of H_2_O and dropped onto a crucible round cover (20 mm diameter). Then, the crucible round cover loaded with BBSN catalysts were dried in a vacuum oven at 50 ℃ overnight before use. The photothermal catalytic CO_2_ reduction tests took place in a 100 mL reaction chamber equipped a top-placed transparent sapphire optical window (40 mm diameter). The chamber temperature could be controlled between room temperature to 240 ℃, and the internal pressure could be controlled in the range from atmospheric pressure to 0.1 MPa. The crucible round cover loaded with 50 mg BBSN catalyst was placed about 1 cm below the center of the sapphire window. 1 mL H_2_O was added to the reaction chamber in advance. A solar simulator was used as the light source to simulate sunlight irradiation and associated with different light band filters. After a certain time interval of photothermal reaction, the solar simulator and temperature control equipment were turned off. The reaction chamber was cooled down to room temperature, and the gas mixture in the reaction chamber was collected with an air bag. Then, the gas mixture was injected into a gas chromatograph equipped with a flame ionization detector (FID) and thermal conductivity detector (TCD) system to measure the proportion of products.

The concentrations of detected C_2_H_4_, CO, and H_2_ were quantified using calibration factors obtained from standard gases. Gas product calculation formula：

*C_i_* $=\frac{A_{i}}{f}$

*Y_i_* (μmol g^-1^ h^-1^)$=\frac{C_{i}\times V_{ves}}{V_{m}\times m_{cat}\times t}$

Where *C_i_* is the concentration of the product, *Y_i_* is the rate of product formation, *A_i_* is the peak area of the product, as determined from the gas chromatography (GC) analysis, *f* is the transformation factor, *V_ves_* is the volume of the reaction vessel (L), *V_m_* is the molar volume of the gas (L/mol), *m_cat_* is the mass of the catalyst (g), *t* is the reaction time (h)

Liquid-phase products were analyzed by ^1^H NMR spectroscopy (Bruker AVANCE NEO 600). No detectable liquid carbon-containing products (e.g., HCOOH, CH_3_OH) were observed within the detection limits of the measurements.

The product selectivity toward C_2_H_4_ was calculated based on the formation rates of carbon-containing products according to the following by equation:

$\text{S}_{\text{C}_{\text{2}}\text{H}_{\text{4}}}\text{=}\frac{\text{Y}_{\text{C}_{\text{2}}\text{H}_{\text{4}}}}{\text{Y}_{\text{C}_{\text{2}}\text{H}_{\text{4}}}\text{+}\text{Y}_{\text{CO}}}$×100%

Where $\text{S}_{\text{C}_{\text{2}}\text{H}_{\text{4}}}$ is the product selectivity of C_2_H_4_, $\text{Y}_{\text{C}_{\text{2}}\text{H}_{\text{4}}}$ and *Y*_CO_ are the formation rates (μmol g^-1^ h^-1^) of produced C_2_H_4_ and CO, respectively.

**Figure S1.** (a) HRTEM images and (b) the corresponding FFT patterns of the selected regions of BBS.

**Figure S2.** TEM image of (a) BBSN-4, (b) BBSN-6, (c) BBSN-10, (d) BBSN-12.

**Figure S3.** The corresponding FFT patterns of the selected regions of BBSN-8.

**Figure S4.** The XPS survey spectra of BBS and BBSN-8.

**Figure S5.** Ni K-edge EXAFS and the curve fit for BBSN-8, shown in k^2^-weighted *k*-space.

**Figure S6.** Ni K-edge EXAFS and the curve fit for NiS, shown in *R*-space.

**Figure S7.** Schematic diagram of photothermal CO_2_ reduction reaction tests.

**Figure S8**. Gas chromatograms derived from (a) FID1, (b) FID2, and (c) TCD detectors of the gas product yielded by photothermal CO_2_ reduction reaction of BBSN-8, (d) ^1^H NMR spectrum of the liquid product yielded by the photothermal CO_2_ reduction reaction of BBSN-8.

**Figure S9.** CO yields and selectivity of CO_2_ reduction on BBS and BBSN under full-spectrum light irradiation.

**Figure S10.** Surface temperature of BBS and BBSN-8 under light irradiation.

**Figure S11.** Mass spectrum of ^13^CO_2_ labeling experiments on BBSN-8.

**Figure S12.** (a) XRD, (b) XPS survey spectra, (c) Ni 2p, (d) Bi 4f spectra of BBSN-8 before and after photothermal catalytic CO_2_ reduction reaction.

**Figure S13.** Charge difference plots for BBSN.

**Figure S14.** Schematic diagram of Bader charge transfer of BBSN.

**Figure S15.** Structural evolution of the Bi−Ni interfacial distance induced by the formation of Bi−S−Ni linkages in BBSN.

DFT calculations reveal that the formation of Bi−S−Ni linkages decreases the Bi−Ni separation from 4.02 to 3.64 Å, which might promote the electron transfer and provide an opportunity to absorb adjacent intermediates towards C−C coupling.

**Figure S16.** Schematic diagram for photoelectron transfer between Bi_19_Br_3_S_27_ and NiS*_x_* after Ni introduction into BBS.

**Figure S17.** UV-Vis-NIR absorption spectra for BBS and BBSN.

**Figure S18.** Tauc plots derived from absorption spectra for determining the band gaps of (a) BBS and (b) BBSN-8.

**Figure S19.** Mott-Schottky plots of (a) BBS and (b) BBSN-8.

**Fig. S20.** Schematics illustration of the electronic band structures for BBS and BBSN-8.

**Figure S21.** Electrochemical impedance plots of BBS and BBSN.

**Figure S22.** PL spectra of BBSN-8 under different temperatures.

**Figure S23.** Zeta potentials plots of BBS and BBSN-8.

**Figure S24.** Nitrogen adsorption/desorption isotherms of BBS and BBSN-8.

**Figure S25.** Simulated configuration for electron density difference diagrams of (a) BBS and (b) BBSN with CO_2._

**Figure S26.** Adsorption energy of CO in BBS and BBSN.

**Tables**

**Table S1.** Actual Ni/Bi ratios for different samples determined by ICP-OES

| Samples | Ni/Bi (wt%) | Ni/Bi (mol) | Samples | Ni/Bi (wt%) | Ni/Bi (mol) |
| --- | --- | --- | --- | --- | --- |
| BBSN-4 | 0.897 | 0.032 | BBSN-10 | 2.586 | 0.092 |
| BBSN-6 | 1.434 | 0.051 | BBSN-12 | 3.032 | 0.108 |
| BBSN-8 | 1.937 | 0.069 |  |  |  |

Note: The actual Ni/Bi weight ratios were obtained from ICP-OES measurements, and the corresponding molar ratios were calculated based on the atomic weights of Ni and Bi.

**Table S2.** EXAFS fitting parameters at the Ni K-edge for various samples

| Samples | Path | CN^a^ | R(Å)^b^ | σ^2^(Å^2^)^c^ | ΔE_0_(eV)^d^ | R factor |
| --- | --- | --- | --- | --- | --- | --- |
| **Ni K-edge** | | | | | | |
| Ni foil | Ni-Ni | 12 | 2.45±0.03 | 0.006 | 6.31 | 0.002 |
| NiS | Ni-S | 6 | 2.37±0.02 | 0.003* | 2.889 | 0.017 |
|  | Ni-Ni | 2 | 2.59±0.02 | 0.003* |  |  |
| NiO | Ni-O | 6 | 2.09±0.008 | 0.007 | -2.927 | 0.007 |
|  | Ni-Ni | 12 | 2.96±0.004 | 0.006 |  |  |
| BBSN-8 | Ni-S | 2.5 | 2.37±0.03 | 0.009 | -2.677 | 0.009 |
|  | Ni-Ni | 2.8 | 2.59±0.03 | 0.008 |  |  |

a: CN values for Ni foil, NiS, and NiO were fixed based on crystallographic structures, while those for BBSN-8 were obtained from EXAFS fitting. b: The amplitude reduction factor (S_0_^2^) was fixed to 0.79 for all fittings. c: The EXAFS fitting was performed in k-range of 3–12 Å^-1^ and R-range of 1–3 Å using k^2^-weighting. The σ^2^ values marked with * were fixed during fitting.

**Table S3.** Summary of the reported data on CO_2_ reduction over various catalysts

| Catalyst | Light source (mW/cm^2^) | C_2_H_4_ Yield (μmol·g^−1^ h^−1^) | C_2_H_4_ Selectivity (%) | Temperature (℃) | Ref. |
| --- | --- | --- | --- | --- | --- |
| Bi_19_Br_3_S_27_/NiS*_x_* | 300 W Xenon lamp | 41.38 | 96.8 | 240 | This work |
| Bi_19_S_27_Br_3_-V_s_ | 300 W Xenon lamp | 17.80 | 49 | 5 | [2] |
| Bi_2_S_3_@In_2_S_3_ | 1.15 W cm^-2^（320 nm < λ < 1100 nm） | 11.81 | 87.2 | 240 | [3] |
| Bi_2_Te_3_/TiO_2-_*_x_* | 300 W Xenon lamp | 15.08 | C_2_H_4_+C_2_H_6_: 44 | 56 | [4] |
| Ga/CoS_2_ | 1200 mW·cm^-2^ | 12.45 | 94.5^a*^ | 93.5 | [5] |
| Co-doped NiS_2_ | 300 W Xenon lamp | 2.5 | 74.3^b*^ | 20 | [6] |
| CuInP_2_S_6_ | AM 1.5G | 20.89 | 56.4 | 176.7 | [7] |
| Pt-TCPP/Bi_3_O_4_Br | 300 W Xenon lamp | 2.64 | ～33^b*^ | 5 | [8] |
| Cu_3_-MOFs | 300 W xenon lamp (λ ＞ 720 nm, 4h) | 18.25 | 36.2 | 90 | [9] |
| Bi-CDs/LDH | 300 W Xe lamp | 1.35 | 12.7 | 109 | [10] |
| PdCo/C_3_N_4_ | 300 W Xenon lamp | 36.3 | 81.5^b*^ | N.R. | [11] |
| Fe_2_O_3_/GR/Bi_2_O_2_S | 300 W Xenon lamp | 2.88 | 14.3 | 25 | [12] |
| Cu/3DOM-In_2_O_3_ | 300 W Xe lamp | 24.31 | 67.55 | 25 | [13] |
| Vs-NiCo_2_S_4_-NF | 300 W Xe lamp | 13.42 | 51.7 | 20 | [14] |
| FeCoS_2_ | 300 W Xe lamp | 20.1 | 82.9 | 25 | [15] |
| Cu^δ+^/CeO_2_-TiO_2_ | 300 W Xe lamp (320 – 780 nm) | 4.51 | 47.5 | 25 | [16] |
| Cu_1_/W_18_O_49_ | 300 W Xe lamp | 4.9 | 72.8^b*^ | 25 | [17] |

a*refers to the reaction in the presence of TEOA, b* refers to the electron selectivity.

**Table S4.** Textural properties of BBS and BBSN catalysts.

| Samples | BET surface area/m^2^ g^−1^ | Pore volume ^a^ /cm^3^ g^−1^ | Avg. pore diameter ^b^ /nm | |
| --- | --- | --- | --- | --- |
| BBS | 18.6341 | 0.0328 | | 8.1573 |
| BBSN | 65.0261 | 0.0757 | | 5.5148 |

^a^ BJH desorption cumulative pore volume.

^b^ BJH desorption average pore diameter.

**References**

[1] W. Jia, R. Xiong, Y. Sun, Y. Xiao, B. Cheng, and S. Lei, “A Well-Designed Hierarchical Bi_19_S_27_Br_3_ Nanorods@SnIn_4_S_8_ Nanosheet Core–Shell S-scheme Heterostructure for Robust Photothermal-Assisted Photocatalytic CO_2_ Reduction,” *Journal of Materials Chemistry A* **12**, (2024): 4513-4524. <https://doi.org/10.1039/d3ta07169c>.

[2] J. Zhao, H. Chen, B. Wang, et al., “Sulfur-Vacancy Induced Asymmetric Active Site for Bi_19_S_27_Br_3_ Nanorods Photocatalyzes CO_2_ Conversion to Ethylene,” *Applied Catalysis B: Environment and Energy* **361**, (2025): 124647. https://doi.org/10.1016/j.apcatb.2024.124647.

[3] K. Yan, D. Wu, T. Wang, et al., “Highly Selective Ethylene Production from Solar-Driven CO_2_ Reduction on the Bi_2_S_3_@In_2_S_3_ Catalyst with In−S_V_−Bi Active Sites,” *ACS Catalysis* **13**, (2023): 2302-2312. https://doi.org/10.1021/acscatal.2c05741.

[4] B. Zhang, C. Zhao, J. Qiao, et al., “Synergistic Engineering of Oxygen Vacancies and Topological Surface States in Bi_2_Te_3_/TiO_2-_*_x_* Heterostructures for Highly Efficient Photothermal‐Driven CO_2_ Photoreduction,” *Energy & Environmental Materials* **0**, (2026): e70258. https://doi.org/10.1002/eem2.70258.

[5] C. An, Y. Nie, J. H. Mei, et al., “Regulating the Electron Distribution to Accelerate the Photocatalytic Reduction of CO_2_ to C_2_H_4_,” *ACS Nano* **19**, (2025): 15912-15923. https://doi.org/10.1021/acsnano.5c01548.

[6] W. Shao, X. Li, J. Zhu, et al., “Metal^n+^-Metal^δ+^ Pair Sites Steer C−C Coupling for Selective CO_2_ Photoreduction to C_2_ Hydrocarbons,” *Nano Research* **15**, (2021): 1882-1891. https://doi.org/10.1007/s12274-021-3789-x.

[7] W. Gao, L. Shi, W. Hou, et al., “Tandem Synergistic Effect of Cu-In Dual Sites Confined on the Edge of Monolayer CuInP_2_S_6_ toward Selective Photoreduction of CO_2_ into Multi-Carbon Solar Fuels,” *Angewandte Chemie International Edition* **63**, (2024): e202317852. https://doi.org/10.1002/anie.202317852.

[8] L. Wang, R. Wang, S. Wei, et al., “Tuning the Band Gap Energy of Cu_x_In_y_S for Superior Photothermocatalytic CO_2_ Conversion to C_2_H_4_,” *Industrial Chemistry & Materials* **3**, (2025): 440-451. https://doi.org/10.1039/d5im00015g.

[9] Y. O. He, W. Y. Zheng, Y. Liu, et al., “Direct Air Capture and Photoconversion of CO_2_ to Ethylene by Defect-Tailored Cu_3_-Based Metal-Organic Frameworks,” *Angewandte Chemie International Edition* **65**, (2026): e16438. https://doi.org/10.1002/anie.202516438.

[10] L. Q. Yu, R. T. Guo, C. Xia, S. H. Guo, J. S. Yan, and Y.F. Liu, “Bismuth-Metal and Carbon Quantum Dot Co-Doped NiAl-LDH Heterojunctions for Promoting the Photothermal Catalytic Reduction of CO_2_,” *Small* **21**, (2025): e2409901. https://doi.org/10.1002/smll.202409901.

[11] C. Huang, X. L. Yu, G. C. Lv, Y. M. Hu, and L. B. Liao, “Nitrogen Vacancies Rich C_3_N_4_ Supported PdCo Catalyst for Selective Photocatalytic CO_2_ Reduction to C_2_H_4_,” *Journal of Materials Chemistry A* **13**, (2025): 34948–34954. https://doi.org/10.1039/D5TA05916J.

[12] Y. Luo, H. R. Han, G. Z. Zhang, Q. Z. Wang, and Y. F. Jia, “Construction of Z-Scheme α-Fe_2_O_3_/Graphene/Bi_2_O_2_S Heterojunction for Visible-Light-Driven Photocatalytic CO_2_ Conversion,” *Separation and Purification Technology* **314**, (2023): 123607. https://doi.org/10.1016/j.seppur.2023.123607.

[13] M. Xu, Q. Y. Zhang, S. P. Wei, et al., “Engineering Heteronuclear Dual-Metal Active Sites in Ordered Macroporous Architectures for Enhanced C_2_H_4_ Production from CO_2_ Photoreduction,” *Angewandte Chemie International Edition* **64**, (2025): e202506072. https://doi.org/10.1002/anie.202506072.

[14] X. Luo, L. Qiao, S. Zhang, et al., “S-Vacancy-Assisted Dual-Sites on NiCo_2_S_4_ for Photoconversion of CO_2_ to Olefiant Gas,” *Applied Surface Science* **601**, (2022): 154184. https://doi.org/10.1016/j.apsusc.2022.154184.

[15] Y. Wu, Q. Chen, J. Zhu, et al., “Selective CO_2_‐to‐C_2_H_4_ Photoconversion Enabled by Oxygen‐Mediated Triatomic Sites in Partially Oxidized Bimetallic Sulfide,” *Angewandte Chemie International Edition* **62**, (2023): e202301075. https://doi.org/10.1002/anie.202301075.

[16] T. Wang, L. Chen, C. Chen, et al., “Engineering Catalytic Interfaces in Cu^δ+^/CeO_2_-TiO_2_ Photocatalysts for Synergistically Boosting CO_2_ Reduction to Ethylene,” *ACS Nano* **16**, (2022): 2306-2318. https://doi.org/10.1021/acsnano.1c08505.

[17] Y. Mao, M. Zhang, G. Zhai, et al., “Asymmetric Cu(I)−W Dual‐Atomic Sites Enable C−C Coupling for Selective Photocatalytic CO_2_ Reduction to C_2_H_4_,” *Advanced Science* **11**, (2024): 2401933. https://doi.org/10.1002/advs.202401933.
